# Lipid Nanostructures for Antioxidant Delivery

**DOI:** 10.3390/antiox11020238

**Published:** 2022-01-26

**Authors:** Elisabetta Esposito

**Affiliations:** Department of Chemical, Pharmaceutical and Agricultural Sciences, University of Ferrara, Via Fossato di Mortara, 19-44121 Ferrara, Italy; ese@unife.it; Tel.: +39-0532-455230

The recent scientific literature has demonstrated the attractive potential of lipid-based nanosystems, for many pharmaceutical applications. Indeed, lipid nanostructures can dissolve drugs with different physical and chemical properties, especially lipophilic active molecules, increasing their bioavailability and efficacy, as well as controlling delivery, while decreasing side effects [1]. The rationale behind using lipid nanosystems is also related to their biocompatibility and scarce toxicity, offering the chance to administer them by different routes. Many studies have demonstrated that the nanoencapsulation strategy enables the protection of active molecules from possible degradation, prolonging their chemical stability [2]. On this matter, the inclusion of antioxidants within lipid nanostructures offers a tremendous opportunity. Indeed, despite their pharmaceutical potential, spanning from free radical formation hindrance to the inhibition of cancer cell proliferation and progression, antioxidant molecules are characterized by poor stability, and are degraded by many environmental factors, such as oxygen, light, high temperature, and humidity [3]. In this regard, lipid-based nanostructures, such as liposomes, ethosomes, transethosomes, solid lipid nanoparticles, and nanostructured lipid carriers, have been recently proposed as innovative delivery systems for antioxidant molecules. In vitro and in vivo studies have demonstrated that antioxidant encapsulation prolongs release kinetics, bioavailability, and antioxidant effects [4]. Accordingly, this special issue reports on four original research papers, supporting interesting results about different aspects.

The first study describes the incorporation of an extract of pepper, of the *Capsicum* species, in liposomes [5]. Apart from their flavoring properties, peppers possess a phytochemical content, characterized by different biological activities. Particularly, their notable anti-inflammatory property is related to the high levels of antioxidant compounds, exerting synergic activity [6]. A methanolic extract was obtained from a new pepper genotype and tested for anti-inflammatory activity. Two kinds of phospholipid vesicles were produced, in order to encapsulate the pepper extract, increasing the bioavailability of its bioactive components. Viability tests, conducted in a human monoblastic leukemia U937 cell line, demonstrated a scarce in vitro cytotoxicity of the pepper extract, containing liposomes. Intracellular reactive oxygen species (ROS) and nitric oxide levels were measured, in order to study the in vitro efficacy of the vesicles in regulating inflammatory responses, demonstrating that the liposomal incorporation significantly reduced ROS levels in extract-treated activated cells. Remarkably, liposomes were able to improve the transport of pepper extract components across the cell membrane, as well as accumulating in the cytoplasm, as demonstrated by liquid chromatograph triple quadrupole mass spectrometer analyses [5].

The second article describes a formulative study, aimed at producing nanoparticles constituted of poly lactic-co-glycolic acid, for SUMOylation-deficient Prdx6 delivery to the eye [7]. This recombinant protein can be employed in the treatment of diseases related to oxidative stress, such as cataractogenesis [8]. The nanoparticles, produced by a water-in-oil-in-water emulsion solvent evaporation technique, were characterized by a spherical shape, with a mean diameter ranging between 50 and 250 nm, and a negative zeta potential, suggesting their suitability for subconjunctival administration. The authors demonstrated that the subconjunctival administration of nanoparticles in the eyes enabled a delay or prevention in the cataract formation, blocking oxidative stress-driven adverse signaling, due to ROS-mediated insult reduction. Particularly, the in vitro and subconjunctival delivery of SUMOylation-deficient Prdx6 nanoparticles allowed for the protection of the lens epithelial cells, derived from human and Prdx6^−/−^-deficient mouse lenses, against oxidative stress, as well as to delay the lens opacity in Shumiya cataract rats. Remarkably, these encouraging results suggest the possibility of employing the nanoparticle to delay cataracts, and in general for oxidative-related pathobiology.

In the third article of this issue, the design and development of ethosomes and transethosomes as topical delivery systems for mangiferin is described [9]. The natural glucosyl xanthone mangiferin can be proposed for skin protection against cutaneous diseases, due to its remarkable antioxidant and anti-inflammatory activity. To improve mangiferin solubility and to promote its permeation through the skin, its inclusion in phosphatidylcholine nanosystems is proposed. Particularly, nanovesicular dispersions, constituted of phosphatidylcholine and ethanol, the so-called “ethosomes”, as well as the same dispersions produced in the presence of surfactants, the so-called “transethosomes”, were produced and characterized [10]. Zeta potential, vesicle size distribution and stability were affected by the vesicle composition. Franz cell studies indicated that the diffusion kinetic of mangiferin was faster in the case of transethosomes, with respect to ethosomes.

In order to investigate the potential of mangiferin-containing nanovesicles, to treat skin disorders associated to pollutants, the antioxidant and anti-inflammatory effects of ethosomes and transethosomes was investigated on human keratinocytes, exposed to cigarette smoke, employed to induce an oxidative and inflammatory status. Remarkably, the results evidenced the capability of mangiferin, loaded in nanovesicular systems, to protect cells from damage. Particularly, the results demonstrated the delivery of mangiferin to the target cell, exploited by ethosomes and transethosomes. The presence of intact vesicles within keratinocytes was further corroborated by transmission electron microscopy analyses, suggesting that both ethosomes and transethosomes were able to pass and deliver their cargo within the cells.

The last paper is a review article about solid lipid nanoparticles and nanostructured lipid carriers, designed for phenolic compound nanoencapsulation. It is well known that natural phenolic compounds possess many beneficial biological effects, due to their antioxidant and anti-carcinogenic potential, allowing for the prevention or treatment of many chronic diseases [11]. Nonetheless, the major drawback associated to phenolic compound use is their low bioavailability, as well as chemical instability, with respect to pH, temperature, and light. To overcome these constraints, lipid-based nanosystems have been proposed. Indeed the strategy of nanoencapsulation in lipid systems allows us to control natural phenolic compound delivery, protecting them from chemical degradation induced by external factors, while improving bioavailability [12]. Particularly, the preparation method’s solid lipid nanoparticles and nanostructured lipid carriers are described, as well as some functionalization approaches. Remarkably, the nanoencapsulation strategy in solid lipid nanoparticles and nanostructured lipid carriers has been demonstrated to increase phenolic compound bioavailability and stability, suggesting their possible use in cosmetic and pharmaceutical products [13].

## References

[1] Puglia C., Pignatello R., Fuochi V., Furneri M.P., Lauro R.M., Santonocito D., Cortesi R., Esposito E. (2019). Lipid Nanoparticles and Active Natural Compounds: A Perfect Combination for Pharmaceutical Applications. Curr. Med. Chem..

[2] Montenegro L. (2017). Lipid-Based Nanoparticles as Carriers for Dermal Delivery of Antioxidants. Curr. Drug Metab..

[3] Attia M., Essa E.A., Zaki R.M., Elkordy A.A. (2020). An Overview of the Antioxidant Effects of Ascorbic Acid and Alpha Lipoic Acid (in Liposomal Forms) as Adjuvant in Cancer Treatment. Antioxidants.

[4] Grgić J., Šelo G., Planinić M., Tišma M., Bucić-Kojić A. (2020). Role of the Encapsulation in Bioavailability of Phenolic Compounds. Antioxidants.

[5] Pappalardo I., Santarsiero A., De Luca M., Acquavia M.A., Todisco S., Caddeo C., Bianco G., Infantino V., Martelli G., Vassallo A. (2021). Exploiting the Anti-Inflammatory Potential of White Capsicum Extract by the Nanoformulation in Phospholipid Vesicles. Antioxidants.

[6] Villa-Rivera M.G., Ochoa-Alejo N. (2020). Chili pepper carotenoids: Nutraceutical properties and mechanisms of action. Molecules.

[7] Chhunchha B., Kubo E., Kompella U.B., Singh D.P. (2021). Engineered Sumoylation-Deficient Prdx6 Mutant Protein-Loaded Nanoparticles Provide Increased Cellular Defense and Prevent Lens Opacity. Antioxidants.

[8] Chhunchha B., Kubo E., Fatma N., Singh D.P. (2017). Sumoylation-deficient Prdx6 gains protective function by amplifying enzymatic activity and stability and escapes oxidative stress-induced aberrant Sumoylation. Cell Death Dis..

[9] Sguizzato M., Ferrara F., Hallan S.S., Baldisserotto A., Drechsler M., Malatesta M., Costanzo M., Cortesi R., Puglia C., Valacchi G. (2021). Ethosomes and Transethosomes for Mangiferin Transdermal Delivery. Antioxidants.

[10] Abdulbaqi I.M., Darwis Y., Khan N.A.K., Assi R.A., Khan A.A. (2016). Ethosomal nanocarriers: The impact of constituents and formulation techniques on ethosomal properties, in vivo studies, and clinical trials. Int. J. Nanomed..

[11] Borges A., de Freitas V., Mateus N., Fernandes I., Oliveira J. (2020). Solid Lipid Nanoparticles as Carriers of Natural Phenolic Compounds. Antioxidants.

[12] Faridi Esfanjani A., Assadpour E., Jafari S.M. (2018). Improving the bioavailability of phenolic compounds by loading them within lipid-based nanocarriers. Trends Food Sci. Technol..

[13] Nunes S., Madureira R., Campos D.A., Sarmento B., Gomes A.M., Pintado M., Reis F., Madureira A.R., Pintado M.M. (2017). Solid lipid nanoparticles as oral delivery systems of phenolic compounds: Overcoming pharmacokinetic limitations for nutraceutical applications. Crit. Rev. Food Sci. Nutr..

